# Liquid Biopsy-Based Metabolomics in Epithelial Ovarian Cancer: Challenges, Methodological Advances and Translational Considerations

**DOI:** 10.3390/diagnostics16131983

**Published:** 2026-06-25

**Authors:** Mariagrazia D’Agostino, Luna Laera, Martina Lanza, Doron Tolomeo, Monica Montopoli, Clelia Tiziana Storlazzi, Gennaro Cormio, Alessandra Castegna, Stefano Miglietta

**Affiliations:** 1 Department of Biosciences, Biotechnologies and Environment, University of Bari Aldo Moro, Via Orabona 4, 70125 Bari, Italy; mariagrazia.dagostino@uniba.it (M.D.); luna.laera@uniba.it (L.L.); doron.tolomeo@uniba.it (D.T.); cleliatiziana.storlazzi@uniba.it (C.T.S.); 2 Department of Experimental Medicine, University of Salento, Via per Monteroni, 73100 Lecce, Italy; martina.lanza@unisalento.it; 3 Department of Pharmaceutical and Pharmacological Sciences, University of Padova, 35131 Padova, Italy; monica.montopoli@unipd.it; 4 Gynecologic Oncology Unit, Istituto di Ricovero e Cura a Carattere Scientifico (IRCCS) Istituto Tumori “Giovanni Paolo II”, Viale Orazio Flacco 65, 70124 Bari, Italy; gennaro.cormio@uniba.it; 5 Department of Interdisciplinary Medicine, University of Bari Aldo Moro, Piazza Giulio Cesare 11, 70124 Bari, Italy

**Keywords:** ovarian cancer, liquid biopsy, metabolomics, cancer reprogramming, biomarkers, precision medicine

## Abstract

Epithelial ovarian cancers (EOCs) histotypes are characterized by marked molecular heterogeneity and limited effectiveness of current screening and monitoring strategies. Earlier identification of tumor-associated alterations may support timely intervention, especially in genetically predisposed or early-onset patient populations. While liquid biopsy approaches have primarily focused on circulating DNA, RNA, and proteins, increasing evidence indicates that cancer-associated metabolic reprogramming generates measurable informative signals in peripheral biofluids. This review summarizes recent progress in liquid biopsy-derived metabolomics in EOCs, covering analytical platforms applied to serum, plasma, urine, and ascites. Recurrent metabolic signatures linked to tumor burden, disease stage, treatment response, and clinical outcome are described, and their significance in discriminating malignant and non-malignant conditions is critically discussed. Collectively, these findings suggest that metabolomics may provide complementary functional information alongside genomic and histopathological profiling. Although its clinical implementation still requires further validation and methodological standardization, ongoing advances in analytical technologies and the integration of high-dimensional metabolic data into machine learning-based frameworks may progressively support the identification of early tumor-associated alterations and contribute to more accurate disease stratification and biologically informed clinical management.

## 1. Introduction

Ovarian cancer (OC) represents one of the most lethal malignancies affecting the female reproductive system, largely due to its asymptomatic onset and the lack of effective strategies for early detection. Current screening approaches remain inadequate: widely used serum markers, such as the ovarian carcinoma antigen CA125, lack sufficient sensitivity and specificity for early-stage disease, with definitive diagnosis still relying on histopathological evaluation, often at advanced stages [1]. Delayed diagnosis not only worsens clinical outcomes but also frequently necessitates aggressive surgical and chemotherapeutic interventions that irreversibly compromise ovarian function [2].

Beyond genetic susceptibility, ovarian physiology is highly sensitive to systemic metabolic cues, hormonal regulation, and environmental influences. Perturbations of these interconnected networks are increasingly recognized as integral components of tumor initiation and progression [3]. At the same time, pharmacological and hormonal interventions used in assisted reproduction and infertility treatments have raised concerns regarding their long-term impact on ovarian homeostasis and cancer risk, underscoring the need for sensitive tools capable of capturing early, functional alterations before overt disease develops [4]. In this context, metabolomics has gained increasing attention as a systems-level approach to investigate cancer-associated metabolic reprogramming through minimally invasive liquid biopsy (LB). By characterizing alterations in lipid, amino acid, nucleotide, and energy metabolism, metabolomic profiling may provide insights into tumor-associated biological processes, disease aggressiveness, and host–tumor interactions, complementing conventional diagnostic and monitoring strategies [5].

This review focuses on recent advances in LB-based metabolomics in EOCs, highlighting how tumor-associated metabolic alterations may improve early diagnosis beyond current screening limitations, refine disease stratification, and ultimately contribute to preserving ovarian function by enabling earlier and more tailored clinical intervention. Importantly, ongoing technical improvements in analytical platforms, including enhanced sensitivity, coverage, and standardization of mass spectrometry (MS)-based workflows, together with advances in data integration and computational analysis, are progressively increasing the translational potential of metabolomic profiling in clinical oncology [6]. Relevant studies were identified through searches in major scientific databases, including PubMed and Scopus, using keywords related to ovarian cancer, liquid biopsy, and metabolomics. In addition, the ClinicalTrials.gov registry, a publicly accessible database of clinical studies, was consulted to identify ongoing and completed trials of potential relevance. Priority was given to recent, peer-reviewed, and clinically relevant publications, while seminal studies and highly influential contributions in the fields of liquid biopsy and metabolomics were also considered when relevant.

## 2. Molecular and Diagnostic Heterogeneity of EOC Histotypes

What is collectively referred to as “ovarian cancer” encompasses a highly heterogeneous spectrum of diseases, in which EOCs account for more than 90% of cases and are distinct from the rest (germ cell, sex cord-stromal, mesenchymal tumors, teratomas, etc.) with respect to origin, etiologic pathways and therapeutic response [7,8]. EOCs comprise a group of neoplasms that are biologically distinct in terms of morphology, site of origin, molecular profile and clinical features (Table 1).

According to the International Agency for Research on Cancer of the World Health Organization (IARC) classification, the major histologic subtypes include high-grade serous carcinoma (HGSC), low-grade serous carcinoma (LGSC), clear cell carcinoma (CCC), endometrioid carcinoma (EC) and mucinous carcinoma (MC) [7,8]. Each subtype arises through specific pathogenic pathways and is associated with distinct implications for risk assessment, prevention strategies, clinical management and metabolic signature. Among these entities, HGSC is by far the most prevalent subtype and accounts for the majority of OC-related deaths. It is now widely recognized as a malignancy that predominantly originates from the epithelium of the distal fallopian tube, arising from precursor lesions such as serous tubal intraepithelial carcinoma (STIC), with subsequent secondary involvement of the ovary [7,9]. In contrast, LGSC, as well as EC and CCC, more commonly arise within the ovary and are frequently associated with pre-existing lesions, such as serous borderline tumors or endometriotic foci [9]. Ovarian MC accounts for a relatively small proportion of cases and poses significant diagnostic challenges, as a substantial fraction of tumors historically classified as primary ovarian mucinous neoplasms is now recognized to represent metastases from other sites, particularly of gastrointestinal origin [10].

The mutational landscape and the molecular genetic features of the tumor play a central role in guiding therapeutic decision-making and in shaping clinical outcomes, as they define biologically distinct subgroups with differential sensitivity to treatment [11]. This genotype-driven approach has become central to precision oncology in OC. In parallel, growing evidence indicates that metabolic reprogramming represents an additional layer of tumor heterogeneity that may further influence disease behavior and therapeutic vulnerability [12]. The following sections summarize the molecular features and current diagnostic limitations of the major EOC histotypes. While the metabolic characterization of EOCs remains less mature than their genomic and pathological classification, the marked biological diversity of these tumors suggests that distinct histotypes may also be associated with specific metabolic programs. Although evidence remains limited for several EOC subtypes, this heterogeneity provides a compelling rationale for investigating histotype-specific metabolic signatures through liquid biopsy-based approaches.

### 2.1. Molecular Landscape of EOC: Diagnostic Relevance and Metabolic Implications

Although EOCs share a common anatomical origin, they are characterized by specific molecular alterations with important diagnostic, prognostic, therapeutic, and metabolic implications.

HGSC is most commonly diagnosed EOC at an advanced stage, with approximately 80% of cases presenting as stage III-IV of FIGO staging (International Federation of Gynecology and Obstetrics), and is frequently associated with elevated serum CA125 levels in more than 90% of patients, despite the lack of specificity of this biomarker, as discussed in a subsequent paragraph [8,13]. HGSC is almost universally characterized by alterations in the tumor protein P53 gene (*TP53*), including expression and mutation, assessed by transcriptional [14] and immunohistochemical analyses [15]. Approximately 15% of patients show germline mutations in *BRCA1* and *BRCA2*, which drive defects in homologous recombination that can cause marked genomic instability [16]. A smaller subset of cases is associated with mutations in *RAD51* paralogs C and D (*RAD51C*/D) and *BRCA1* interacting DNA helicase 1 (*BRIP1*) [17]. These features carry important prognostic and therapeutic implications: tumors with homologous recombination deficiency tend to be more sensitive to platinum-based chemotherapy [18] and the use of DNA damage repair with poly-ADP-ribose polymerase (PARP) inhibitors as maintenance therapy has been shown to improve progression-free survival, with the greatest benefit observed in patients harboring BRCA mutations [19,20].

LGSC follows a relatively indolent clinical course and tends to present at an earlier stage [21]. At the molecular level, this subtype is frequently driven by alterations in the mitogen-activated protein kinase (MAPK) signaling pathway, including mutations in genes such as *KRAS*, *NRAS*, *BRAF* [22] and typically retains wild-type *TP53* [23].

CCC is often a unilateral tumor with clear eosinophilic cells, occasionally associated with paraneoplastic hypercalcemia and venous thromboembolic events [8]. CCC is frequently characterized by inactivating alterations in the tumor suppressor AT-rich interaction domain 1A gene (*ARID1A*) [24,25] and mutations in phosphatidylinositol-4,5-bisphosphate 3-kinase catalytic subunit alpha gene (*PIK3CA*), alongside with *ARID1A* loss [26], whereas *KRAS* and *TP53* gene mutations and mismatch repair deficiency are very rare [27].

EC is frequently diagnosed at an early stage and is associated with favorable outcomes [8]. It commonly arises from ovarian endometriosis, with a similar mutation burden [28] and displays heterogeneous molecular alterations involving the WNT/*CTNNB1* (β-catenin), Phosphatidylinositol-4,5-Bisphosphate 3-Kinase (PI3K), MAPK and SWI/SNF pathways, with occasional mismatch repair deficiency or DNA Polymerase Epsilon Catalytic Subunit (*POLE*) mutations [29,30].

MC is defined by intestinal-type differentiation and is commonly diagnosed as a disease confined to the ovary, a feature associated with favorable outcomes at early stages [8]. At the molecular level, early genetic events frequently include *CDKN2A* copy-number loss and *KRAS* mutations, whereas *ERBB2*/HER2 amplifications are more often observed in biologically advanced tumors with *TP53* alterations [10].

According to the IARC classification and contemporary literature, the EOCs diagnosis relies on an integrated, stepwise approach in which histological morphology constitutes the primary and indispensable element, subsequently refined by immunohistochemistry and by molecular analyses performed mainly by modern omics techniques [8,31]. Each histological subtype is defined by characteristic architectural and cytological features, reproducible immunophenotypic profiles, and recurrent genetic alterations that facilitate discrimination among tumor entities (Table 1). Importantly, such molecular characterization has facilitated the development of increasingly tailored therapeutic strategies, supporting the implementation of targeted treatments within a precision oncology paradigm [32]. These genomic alterations predominantly involve genes related to DNA damage response, chromatin remodeling, and key oncogenic signaling pathways, rather than genes directly encoding metabolic enzymes [31]. Consequently, tumor metabolism is largely shaped by downstream functional effects of oncogenic transformation rather than by primary metabolic gene mutations. *TP53* alterations have been associated with enhanced glycolysis, lipid biosynthesis, and activation of the mevalonate pathway [33,34,35], while homologous recombination-deficient cells, including those harboring *BRCA1/2* mutations, display metabolic adaptations characterized by increased reliance on oxidative metabolism, NAD+ homeostasis, and redox-regulating pathways that sustain DNA repair processes [36,37]. *ARID1A*-deficient cells exhibit a marked dependence on glutamine metabolism through upregulation of glutaminase (GLS1), resulting in increased glutamine utilization and tricarboxylic acid cycle activity [38]. Similarly, activation of the PI3K/AKT/mTOR pathway promotes anabolic metabolism by enhancing glucose uptake, lipid synthesis, and nucleotide biosynthesis [39]. In contrast, *KRAS*-driven tumors are characterized by metabolic programs that support glutamine utilization and biosynthetic growth [40].

In this context, emerging LB-based approaches, including metabolomic profiling of blood and other biofluids, extend tumor characterization by capturing dynamic metabolic reprogramming, tumor microenvironment (TME) interactions, and disease evolution. Although not currently incorporated into routine diagnostic criteria, these methodologies could generate complementary data with significant translational potential for diagnostic assessment, disease monitoring, risk stratification, and advancing the understanding of tumor crosstalk [41].

### 2.2. Current EOC Screening Limitation

Despite the advances in integrated histopathological, immunophenotypic, and molecular diagnostics, translating such detailed tumor characterization into effective strategies for early detection and population-level screening of EOCs remains limited. CA125 is a cleavage-derived epitope of the transmembrane protein encoded by the mucin 16 gene (*MUC16*), an extremely high-molecular-weight mucin characterized by a large, highly glycosylated extracellular domain that can be released into the circulation through proteolytic cleavage. Seminal studies have shown that longitudinal changes in serum CA125 reflect the clinical course of EOC and that the antigen is expressed in epithelia of coelomic origin and their derivatives, providing a biological rationale for its clinical use [42,43]. However, CA125 expression is neither tumor-specific nor uniformly elevated in all EOC subtypes, resulting in both false positives (elevation in benign or non-ovarian conditions) and false negatives (absence of elevation in some early stages) that undermine test reliability [44]. The combination of CA125 with human epididymis protein 4 (HE4), together with menopausal status in algorithms such as the Risk of Ovarian Malignancy Algorithm, improves discrimination between benign and malignant adnexal masses but does not constitute an effective strategy for population-based screening [44,45]. Even though HE4 levels increase with advancing age, age-adjusted algorithms, rather than those based solely on menopausal status, may provide more accurate risk stratification when CA125 and HE4 are used in combination [44].

Beyond its role as a circulating biomarker, *MUC16* plays an active role in tumor biology, modulating cell–cell and cell–matrix interactions, promoting mesothelin-mediated adhesion, facilitating peritoneal dissemination, and contributing to immune evasion, all of which increase molecular heterogeneity and complicate biomarker detection across tumor subtypes and stages [46]. Recent evidence indicates that assays targeting specific glycovariants of CA125, which reflect tumor-specific glycosylation changes, significantly enhance diagnostic sensitivity and specificity compared to conventional CA125 immunoassays, particularly for differentiating malignant from benign masses [47].

In addition to biomarker-related challenges, the imaging-based approaches commonly used in screening pose significant limitations. Transvaginal ultrasound is widely employed in OC screening trials because it can detect abnormalities in ovarian volume and morphology; however, it performs poorly at distinguishing benign from malignant lesions and has a low positive predictive value when used as a standalone modality [48]. Moreover, many aggressive OCs originate in anatomical sites such as the fimbriae of the fallopian tubes or remain below the ultrasound resolution threshold until metastatic spread has occurred, leading to false-negative results in early-stage disease [48].

Collectively, these limitations, including the marked biological heterogeneity of OC and the largely asymptomatic nature of early-stage disease, provide a compelling explanation for the failure of current screening strategies to achieve meaningful reductions in OC-specific mortality. This body of evidence underscores the pressing need for next-generation screening strategies that move beyond single-aspect testing and instead integrate novel LB-based approaches, enabling not only earlier detection but also dynamic disease monitoring and, ultimately, improved patient survival.

## 3. Liquid Biopsy in Clinical Practice

Current oncological practices are transitioning toward personalized medicine, a model in which clinical strategies are tailored to the unique genomic and molecular landscape of the patient’s malignancy [49]. In this context, LB has been established as a high-resolution and multidimensional diagnostic modality. By enabling the qualitative and quantitative analysis of circulating tumor-derived biomarkers across various biofluids, LB provides a high-resolution tool for monitoring neoplastic progression [50]. LB offers significant advantages over conventional tissue biopsy techniques, although it is currently considered a complementary diagnostic tool rather than a definitive replacement for initial histological diagnosis [51].

The solid tissue biopsy presents substantial limitations; its invasive nature precludes frequent sampling, and a single core biopsy often provides an incomplete representation of intra-tumoral and microenvironmental heterogeneity [52]. Conversely, LB provides an integrative and holistic molecular characterization of the tumor genomic landscape. This longitudinal perspective enables clinicians to evaluate therapeutic efficacy and detect the emergence of acquired resistance mechanisms at an early stage [53].

LB demonstrates high sensitivity for detecting minimal residual disease following surgical resection, a task that conventional imaging modalities (e.g., Computed Tomography or Magnetic Resonance Imaging) cannot resolve. By identifying molecular recurrence several months before clinical relapse, LB facilitates earlier therapeutic intervention [54,55].

Finally, the rapid turnaround time of emerging biofluid-based assays minimizes operational overhead and streamlines the deployment of targeted therapies tailored to the patient-specific molecular profile [56,57].

### Current LB Targets and Sampling

LB is based on the advanced detection of a heterogeneous array of cancer-related analytes shed into body fluids and categorized by their biological features. The nucleic acid compartment represents the core of this method, most notably circulating tumor DNA (ctDNA), a subfraction of cell-free DNA (cfDNA) released through the apoptosis or necrosis of neoplastic cells. It enables the mapping of genomic alterations, including single-nucleotide variants, insertions/deletions, copy number variations, and epigenetic modifications [58].

Concurrently, the analysis of the circulating transcriptome, including mRNAs, microRNAs (miRNAs), circular RNAs (circRNAs), and other non-coding RNAs, reflects the dynamic transcriptional programs and active molecular signatures inherent to the tumor mass [59,60,61].

Moving beyond circulating nucleic acids, LB can also interrogate cellular and vesicular components that provide complementary information on tumor biology. These include circulating tumor cells (CTCs), circulating cancer-associated fibroblasts (cCAFs), and tumor-educated platelets (TEPs), which may reflect metastatic dissemination, tumor–microenvironment interactions, and systemic responses to cancer [62,63,64,65]. Extracellular vesicles (EVs), particularly exosomes, further contribute to LB analyses by carrying tumor-derived proteins, nucleic acids, and other biomolecules released by viable cells, thereby representing an additional source of potentially informative biomarkers [66,67].

**Figure 1:**
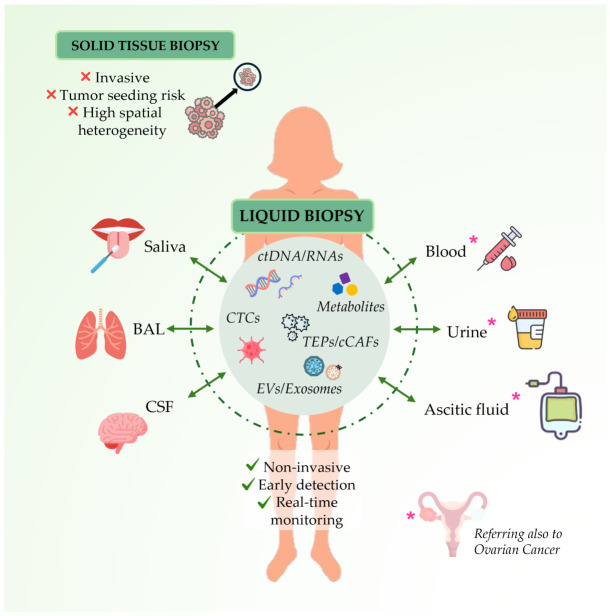
Comparative analysis between solid tissue biopsy and LB. Solid tissue biopsy is traditionally characterized by its invasiveness, potential risk of tumor seeding, and limitations due to high spatial heterogeneity. In contrast, LB offers a non-invasive alternative, enabling early detection and real-time monitoring of disease progression. Beyond blood and urine, various biological fluids such as saliva, BAL (bronchoalveolar lavage), CSF (cerebrospinal fluid), and notably ascitic fluid in ovarian cancer, serve as sources for clinical biomarkers. These include ctDNA/RNAs, CTCs, EVs/Exosomes, TEPs, cCAFs, and several metabolites. Pink asterisks refer specifically to OC.

LB can be applied to different biological matrices, with peripheral blood remaining the most widely used source owing to its minimal invasiveness and standardized collection procedures. Plasma and serum are the preferred specimens for most liquid biopsy applications and are particularly relevant for metabolomic analyses, as they contain a broad spectrum of tumor- and host-derived biomolecules, including oncometabolites [68,69,70]. Beyond blood-derived samples, alternative biofluids such as urine and saliva have also been investigated because of their completely non-invasive collection and potential utility for longitudinal disease monitoring and biomarker discovery [71,72]. Proximal fluids may provide a more direct representation of the local tumor microenvironment by overcoming physiological barriers that can limit the detection of tumor-derived signals in blood. Examples include cerebrospinal fluid, pleural effusions, bronchoalveolar lavage, ascitic fluid, and cystic fluid, which can contain enriched genomic and metabolic biomarkers and may complement blood-based liquid biopsy approaches (Figure 1) [73,74,75,76,77].

LB integrates multiple high-sensitivity analytical platforms designed to isolate and profile oncogenic biomarkers present at infinitesimal concentrations within biological matrices. For nucleic acid analysis, polymerase chain reaction (PCR)-based methodologies, such as digital PCR and droplet digital PCR, enable the absolute quantification of rare variants with detection limits [78]. To achieve a more comprehensive genomic overview, Next-Generation Sequencing (NGS) platforms facilitate extensive profiling, ranging from targeted gene panels to whole-exome or whole-genome sequencing, which can be combined with advanced epigenetic and fragmentomic sequencing to infer the tumor tissue of origin [79,80].

The isolation and characterization of CTCs remain technically challenging because of their extreme rarity in peripheral blood. Current enrichment strategies exploit either physical properties, such as size and density, or immunoaffinity-based approaches targeting tumor-specific surface markers. Advances in CTC enrichment have improved their applicability for downstream molecular analyses and functional studies, supporting their potential role in precision oncology [81,82,83]. Concurrently, the analysis of EVs and exosomes has transitioned from classical ultracentrifugation to size-exclusion chromatography and microfluidic platforms, which offer better standardization [84,85]. The proteomic and lipidomic cargo of these vesicles can then be characterized using high-resolution optical and Surface-Plasmon Detection technologies, generating precise molecular fingerprints that enhance diagnostic specificity within the multi-omics LB framework [86,87].

## 4. Metabolomics of LB in Cancer

The molecular landscape described above can be further complemented by proteomic and metabolomic approaches, which capture the functional consequences of tumorigenesis. Increasingly, these data are being integrated into multi-omics platforms combining genomic, transcriptomic, proteomic, and metabolic information to improve cancer detection and characterization [88,89]. The following section focuses on the role of metabolomics within the liquid biopsy framework, highlighting its biological rationale, clinical relevance, and analytical applications.

### 4.1. Metabolism Deregulation in Cancer

Metabolomics is a scientific discipline dedicated to the systematic identification and quantification of the complete set of metabolites in an organism or a biological sample. Within the hierarchy of the “-Omics” sciences, it represents the final stage, positioned downstream of genomics, transcriptomics, and proteomics. Since metabolites are the end products of cellular activity, metabolomics provides a direct, real-time “functional readout” of an individual’s physiopathological state, accurately reflecting alterations induced by disease.

The transformation of a healthy cell into a malignant one entails extensive metabolic alterations; this is not merely an accelerated version of normal metabolism, but rather a system that has been completely “rewired” to sustain the demands of uncontrolled cellular proliferation. Metabolic reprogramming is the comprehensive reprogramming of biochemical pathways and cellular metabolic fluxes and is currently recognized as a fundamental hallmark of cancer. This condition is not a simple byproduct of the disease, but an active adaptation orchestrated by mutations in key regulatory genes, such as oncogenes of the *MYC* and *RAS* family or the loss of tumor suppressors (e.g., *TP53*, *PTEN*), which force the cell to adopt an aberrant metabolic phenotype [90,91,92]. It enables malignant cells to sustain uncontrolled proliferation, survive in hostile microenvironments, and promote metastatic progression [93,94]. Plastic metabolic alterations in cancer cells are activated to support the tumor through several mechanisms.

Metabolism is critical to tumor cell proliferation. Unlike normal cells, cancer cells convert glucose into lactate even in the presence of oxygen, a phenomenon known as the Warburg effect [95]. Although it is less efficient in terms of ATP yield per glucose molecule, this metabolic pathway is extremely rapid. It provides the biosynthetic intermediates necessary for the synthesis of proteins, nucleic acids, and lipids, which are essential for cellular duplication [96].

Metabolic reprogramming is also exerted through the synthesis of oncometabolites, which sustain the malignant phenotype [97]. Certain genetic mutations lead to the accumulation of oncometabolites, such as 2-hydroxyglutarate, succinate, and fumarate [98]. These molecules modulate epigenetic activity by altering gene expression to favor a malignant phenotype [99]. Furthermore, they suppress DNA repair mechanisms, thereby increasing genomic instability, which accelerates tumor evolution [100]. These same metabolites can regulate gene transcription through chemical histone modifications, such as lactylation, induced by lactic acid accumulation [101].

Metabolism also facilitates adaptation to the TME. The TME can be defined as a complex and dynamic ecosystem surrounding the tumor, composed not only of malignant cells but also a variety of non-cancerous cells, blood vessels, signaling molecules, and the extracellular matrix. It is frequently characterized by extreme conditions to which tumor cells must adapt, such as hypoxia, acidification, and nutrient deprivation; still, it can also serve as a supportive environment for tumor growth. This occurs through intercellular communication mechanisms that can promote immune escape, metastasis, and drug resistance [102,103,104]. Metabolism represents the mechanism by which the TME is modified to favor the tumor. For instance, lactate excretion acidifies the microenvironment, thereby facilitating tissue invasion and promoting the epithelial–mesenchymal transition, which endows cells with migratory capabilities [105]. In OC, the release of N-acetylaspartate (NAA) into the TME promotes the acquisition of a pro-tumorigenic phenotype in tumor-associated macrophages [106]. The reprogramming of lipid metabolism, specifically fatty acid oxidation (FAO), enables cells to survive under hypoxic and nutrient-deprived conditions by enhancing resistance to metabolic stress [107,108]. Furthermore, the accumulation of cholesterol and other metabolites within the microenvironment can induce CD8^+^ T-cell exhaustion, thereby preventing the immune system from mounting an effective anti-tumor response [109]. Metabolic reprogramming is also intrinsically linked to the tumor’s ability to develop resistance to drugs and conventional therapies, enabling cells to activate alternative metabolic pathways to bypass the effects of treatment.

Metabolic reprogramming in cancer cells also extends to amino acid metabolism, which is altered to meet critical energetic and biosynthetic demands. For instance, many tumors become ‘glutamine-addicted’ to fuel the tricarboxylic acid cycle and maintain redox homeostasis, thereby promoting drug resistance [110]. Some tumors upregulate de novo serine synthesis, which is essential for one-carbon metabolism and the production of glycine and cysteine, ultimately sustaining glutathione levels [111]. Furthermore, reprogramming of branched-chain amino acid metabolism is pivotal; it not only contributes to energy production but also activates mTORC1, which directly stimulates tumor biomass expansion [112].

Finally, it has been demonstrated that metabolic reprogramming paves the way for metastasis by generating pre-metastatic niches. In these niches, secreted metabolites, often transported via exosomes, communicate with distant tissues to establish a hospitable environment before the arrival of CTCs [113]. Furthermore, various metabolites have been found to play a critical role in stimulating angiogenesis, thereby ensuring a continuous supply of nutrients to the growing tumor [114].

### 4.2. LB-Based Metabolomics: General Considerations and Limitations

Metabolomics provides a functional layer of information that complements genomic, transcriptomic, and proteomic analyses by capturing the downstream consequences of cellular activity [115,116,117,118]. Through the identification of alterations in biochemical pathways, oncometabolites, and signaling metabolites, metabolomic profiling can reveal disease-associated metabolic states and provide insights into the pathophysiology of complex disorders [119,120,121]. In addition, the characterization of microbiota-derived metabolites and the application of isotope-tracing approaches contribute to the investigation of host–microbiota interactions, metabolic fluxes, and adaptive cellular responses under pathological conditions [122,123].

Within the liquid biopsy framework, metabolomics enables the analysis of circulating metabolic alterations associated with tumor development, progression, and treatment response. Because metabolites represent the downstream products of multiple biological processes, the circulating metabolome may capture both tumor-intrinsic features and systemic host responses, including interactions with the TME, lifestyle factors, and the gut microbiome [116,124].

Regarding therapeutic approaches, metabolic profiles enable the assessment of treatment efficacy and the early detection of recurrence almost immediately, overcoming the interpretability limits of other markers for therapeutic monitoring [125,126]. Due to their short half-lives, metabolites capture a “dynamic window” of tumor activity, enabling near-real-time monitoring of treatment response and the early identification of emerging drug resistance [127]. Finally, integrating metabolic and transcriptomic data has demonstrated great potential in discovering altered metabolic pathways that can serve as novel therapeutic targets.

From an analytical standpoint, metabolomic profiling can be performed using relatively small biofluid volumes and relies on established analytical platforms, supporting its investigation as a complementary liquid biopsy approach. Nevertheless, the interpretation and clinical translation of metabolomic data remain challenging. Unlike tumor-specific genomic alterations, circulating metabolite levels are highly dynamic and may be influenced by pre-analytical procedures, sample handling, fasting status, diet, medication exposure, body mass index, menopausal status, hormonal treatments, microbiome composition, and systemic inflammatory or metabolic conditions. Moreover, many metabolic alterations reflect convergent biological responses and are therefore not necessarily specific to a particular tumor type, potentially limiting diagnostic specificity. Consequently, the translational value of LB-based metabolomics may reside less in isolated metabolites than in multidimensional signatures capturing coordinated pathway-level perturbations. In this perspective, metabolomic profiles should be interpreted as functional phenotypes reflecting the integrated consequences of tumor-intrinsic alterations, host responses, systemic physiology, and environmental exposures. Additional challenges include the accurate annotation and quantification of metabolites, variability across analytical platforms and study designs, and limited reproducibility between independent studies. These issues have been recognized by the Metabolomics Quality Assurance and Quality Control Consortium (mQACC), which has highlighted the need for standardized reference materials, harmonized quality assurance and quality control procedures, and improved cross-laboratory comparability to enhance data robustness and reproducibility [128]. Consequently, the successful clinical implementation of LB-based metabolomics will require rigorous standardization of pre-analytical and analytical workflows, implementation of harmonized QA/QC strategies, robust metabolite identification, and validation in large independent cohorts before routine clinical adoption can be achieved.

### 4.3. Technological Options for Metabolomics Applied to LB

Mass spectrometry (MS) and nuclear magnetic resonance (NMR) spectroscopy are the two principal analytical platforms used in metabolomics, offering complementary strengths and limitations. Among them, MS represents the cornerstone of modern metabolomics owing to its high sensitivity and ability to identify and quantify large numbers of metabolites based on their mass-to-charge ratio (*m*/*z*). To address the complexity of biological samples and reduce matrix effects, MS is commonly coupled with separation techniques. Liquid chromatography (LC-MS) is widely used for the analysis of polar and thermolabile compounds, including amino acids, nucleosides, and lipids; gas chromatography (GC-MS) is particularly suited for volatile or derivatized metabolites and provides high analytical reproducibility; whereas capillary electrophoresis (CE-MS) enables the characterization of highly polar and charged metabolites using minimal sample volumes. MS-based metabolomics can be performed using either untargeted or targeted approaches. Untargeted analyses are primarily employed during biomarker discovery to characterize the metabolome without predefined assumptions, often using acquisition strategies that maximize metabolite coverage [116]. In contrast, targeted approaches focus on the accurate quantification of selected metabolites through the use of internal and external standards, providing greater analytical precision and robustness [116].

Technological advances have expanded the capabilities of MS-based metabolomics through innovative approaches such as MS imaging, real-time tissue analysis, and single-cell metabolomics. MS imaging, often performed using Desorption Electrospray Ionization (DESI), enables the visualization of metabolite spatial distribution within tissue sections while preserving morphological information, thereby facilitating the investigation of tumor heterogeneity [129]. Rapid Evaporative Ionization MS can be integrated with electrosurgical devices to provide near real-time metabolic assessment of tumor margins during surgery [130]. At the highest level of resolution, single-cell metabolomics allows the characterization of individual cancer cells, revealing intratumoral and circulating tumor cell heterogeneity and providing insights into mechanisms underlying metastasis and drug resistance [131].

NMR spectroscopy identifies and quantifies metabolites by exploiting the magnetic properties of atomic nuclei, providing structural and quantitative information on a wide range of compounds. Because NMR is not coupled to separation techniques, biofluid samples generally require the removal of macromolecules through procedures such as methanol precipitation or ultrafiltration, although some metabolite loss may occur due to protein binding. Nevertheless, sample preparation remains relatively simple compared with many MS-based workflows, and the technique is non-destructive, allowing samples to be preserved for subsequent analyses [126]. Like MS, NMR supports both research and clinical applications. Metabolic signatures identified ex vivo can often be investigated in vivo using Magnetic Resonance Spectroscopic Imaging, facilitating clinical translation. In addition, High-Resolution Magic Angle Spinning (HR-MAS) NMR enables the analysis of intact tissue specimens without homogenization, preserving structural and biochemical information while providing detailed metabolic characterization [132,133].

MS and NMR possess distinct analytical characteristics and are therefore considered highly complementary technologies in metabolomics. NMR does not require prior chromatographic separation, minimizes sample manipulation, preserves the specimen for subsequent analyses, and provides highly reproducible data with absolute quantitative capabilities. These features facilitate robust metabolite identification and inter-laboratory comparability. In contrast, MS offers substantially greater sensitivity, enabling the detection of low-abundance metabolites across a broader dynamic range and with higher analytical resolution [134]. Coupling MS with separation techniques such as LC or GC further enhances metabolite coverage and reduces matrix complexity.

Because of these complementary strengths, MS and NMR are frequently used in combination. MS is commonly employed during the discovery phase to generate comprehensive metabolic profiles and identify candidate biomarkers, whereas NMR or targeted MS approaches are subsequently used to confirm metabolite identity and obtain accurate quantitative measurements [134,135]. The integration of these platforms combines the sensitivity and coverage of MS with the reproducibility and structural information provided by NMR, enabling a more comprehensive characterization of metabolic alterations associated with disease. Nevertheless, methodological variability across analytical platforms and workflows remains a significant challenge, highlighting the importance of harmonized QA/QC procedures and standardized reporting to improve reproducibility and facilitate clinical translation.

## 5. Recent Findings in LB-Derived Molecular Profiling in EOCs

EOCs are typically asymptomatic at early stages and, since effective screening tools are lacking, most patients are diagnosed with advanced disease. Compared to conventional tissue biopsy, which is often constrained by procedural risks and the inability to capture tumor spatial heterogeneity, analyzing neoplastic biomarkers in biological fluids could facilitate the dynamic surveillance of ovarian tumor evolution and mechanisms of chemoresistance [136]. Additionally, from a future research perspective, it is interesting to hypothesize how LB might complement imaging in OC diagnostics to optimize exploratory workflows. As a theoretical concept, LB may serve as a molecular guide, directing clinicians toward targeted imaging based on specific circulating biomarkers. However, it must be emphasized that current clinical evidence for such integration remains strictly limited and speculative, as no clinical trials have yet validated the use of metabolomic biomarkers to direct surgical triage or imaging choice in routine practice. Primarily, conventional imaging often lacks the resolution to detect microscopic peritoneal seeding. LB may have the potential to provide evidence of active disease even when imaging is inconclusive or negative for macroscopic spread. This synergy could facilitate precision imaging by guiding radiologists toward specific peritoneal compartments. Furthermore, it might enhance risk stratification, enabling a more informed selection between standard Computed Tomography (CT) and high-sensitivity Diffusion-Weighted MRI (DW-MRI) for the detection of occult carcinomatosis.

Furthermore, the detection of malignancy-associated biomarkers via LB might help define the ‘molecular suspicion’ requisite for escalating diagnostic protocols, that is, the molecular evidence justifying the utilization of high-resolution Ovarian-Adnexal Reporting and Data System (O-RADS) assessment through ultrasonography or MRI [137,138,139]. This would ensure a comprehensive morphological characterization of adnexal masses prior to surgical intervention, a step particularly vital for patients presenting with indeterminate findings on baseline imaging. Moreover, a high molecular tumor burden identified by LB may streamline the referral process for CT-based mapping or diagnostic laparoscopy to calculate the Fagotti Score [140]. Such stratification is instrumental in identifying patients who may be suboptimal candidates for primary debulking, thereby preventing futile primary surgeries and prioritizing those who would derive greater benefit from neoadjuvant chemotherapy. In perspective, LB should not be conceptualized as a substitute for traditional imaging in OC, but as a molecular compass informing the clinical trajectory. By offering early insights into both malignancy and systemic tumor load, LB might facilitate a more judicious and cost-effective deployment of advanced modalities, such as DW-MRI and laparoscopy-based scoring, ultimately refining the selection criteria for primary cytoreduction.

Ovarian CTCs represent a biologically relevant but technically challenging LB component, owing to their extremely low frequency in peripheral blood [141]. Multiple enrichment strategies have been explored, targeting epithelial markers or exploiting physical cell properties to capture tumor heterogeneity and early dissemination. However, epithelial-to-mesenchymal transition, common in OC, reduces epithelial marker expression and limits the performance of immunoaffinity-based approaches [142]. CellSearch^®^ is currently the only FDA-approved platform for CTCs detection in EOCs, based on cell surface marker expression EpCAM or MOC31/CK/EGFR [143]. CTC enumeration using this system has shown limited correlation with clinical outcomes, highlighting the need for more sensitive and functionally informative detection strategies [144].

In EOC, ctDNA represents one of the most extensively investigated liquid biopsy analytes, enabling the mapping of pathognomonic mutations, such as *TP53* variants found in nearly all HGSC, as well as the assessment of epigenetic methylation profiles via innovative assays designed for the differential diagnosis of pelvic masses [145]. The molecular characterization of the *ERCC1* transcript within CTCs has emerged as a superior indicator compared to traditional immunohistochemical analysis for the early prediction of platinum resistance [146].

Among RNA-based biomarkers, miRNAs have attracted particular interest in early detection owing to their relative stability in circulation compared with mRNAs and proteins. Distinct miRNA expression patterns have been associated with clinical outcomes, with overexpression of miR-21, miR-221, miR-141, and miR-429, and reduced levels of miR-200c, miR-145 and miR-199a, correlating with poorer overall survival [144,147]. MiRNA profiling is the most commonly performed using microarray-based platforms or NGS, each offering complementary advantages in terms of throughput, cost, and discovery potential.

Additional classes of non-coding RNAs, including long non-coding RNAs (lncRNAs) and circRNAs, are emerging as potential diagnostic and prognostic biomarkers, although clinical validation remains limited. circRNAs are of particular interest because of their enhanced stability in peripheral blood, whereas the diagnostic performance of lncRNAs has yet to be clearly established [144].

The bioanalytical framework extends further to metabolomics and the study of TEPs [148,149]. These investigations are no longer confined to the hematic compartment but are expanding toward proximal LB. The utilization of uterine lavage and cervical samples exploits the migration of neoplastic cells from the Fallopian tubes into the uterine cavity to intercept STIC with higher sensitivity than plasma-based methods [150,151], while urinalysis represents a completely non-invasive frontier for screening miRNAs and metabolites [152,153].

EVs, particularly exosomes, represent another important component of LB approaches, as their abundance is increased in the serum of patients with OC, and they carry tumor-derived proteins and RNAs. Exosomal markers such as claudin-4, as well as combined assessment of exosomal miRNAs with established serum biomarkers including CA125 and HE4, have shown improved diagnostic performance. Finally, transcriptomic profiling of TEPs by RNA sequencing has emerged as a highly specific and reproducible strategy for OC detection across different populations, further expanding the scope of LB applications in this disease.

The clinical relevance of LB in OC is further supported by several ongoing and completed clinical trials (Table S1). Most studies focus on ctDNA-based approaches for diagnosis, minimal residual disease detection, and treatment monitoring using peripheral blood, while additional LB components, including CTCs, extracellular vesicles, and circulating RNAs, are also being investigated.

**Figure 2:**
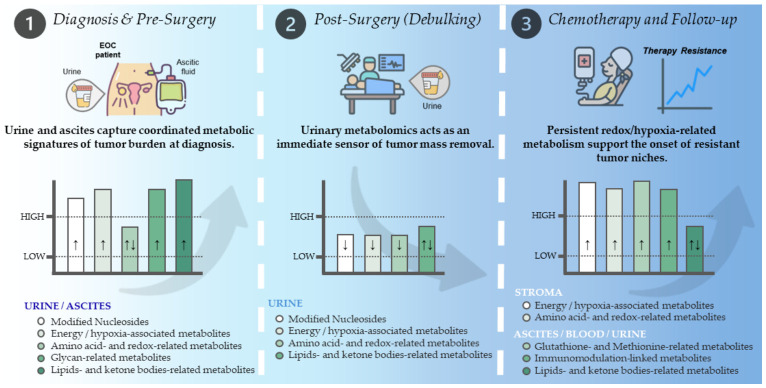
Longitudinal metabolomics as a dynamic sensor of tumor burden, surgical response, and chemoresistance in EOC. Arrows indicate consistent direction of change relative to non-malignant controls or post-surgical baseline. Modified nucleosides: pseudouridine, N4-acetylcytidine, urate-3-ribonucleoside, 5-phosphoribosylamine. Energy/hypoxia-associated metabolites: succinate, taurine, 3-hydroxybutyric acid, acylcarnitines, palmitoylcarnitine. Amino acid- and redox-related metabolites: L-histidine, glutamate, glycine, cysteine/cystine, N-acetylglutamine, phenylacetyl glutamine, hippuric acid, creatinine, lipoamide. Glycan-related metabolites: 3-sialyllactose, 3-sialyl-N-acetyllactosamine. Lipids and ketone bodies-related metabolites: phosphatidylcholines, lysophosphatidylcholines, phosphatidylethanolamines, lysophosphatidylethanolamines, sphingomyelins, cholesteryl esters, ceramides, triacylglycerols, diacylglycerols. Immunomodulation-linked metabolites: lactate, kynurenine, tryptophan, N-acetylaspartate, citraconic acid and itaconate-related metabolites. Glutathione- and Methionine-related metabolites: glutathione, methionine, cysteine/cystine, glutamate, glycine.

Alongside the classical molecular drivers described above, the role of metabolic reprogramming is increasingly recognized as a functional contributor to tumor progression, invasiveness, and therapeutic resistance [154]. In this context, LB extends beyond the analysis of circulating tumor components (ctDNA, microRNA, exosomes, CTCs, TEPs, etc.) to encompass systemic phenotypic readouts of disease biology. Metabolomic profiling of biofluids has therefore emerged as a promising approach to identify OC-associated metabolic signatures [5]. Blood (serum or plasma) is the most commonly used matrix and reflects systemic metabolism, although tumor-derived signals may be diluted [13]. Urine is a non-invasive, biochemically stable matrix suitable for large-scale screening [155]. Proximal fluids, such as ascites and ovarian cyst fluid, provide more direct information on the TME and peritoneal disease progression [13,136]. As such, peripheral metabolomics provides a unifying framework to interrogate therapy resistance, disease progression, treatment response, molecular heterogeneity, and the influence of systemic risk factors. With respect to disease progression and survival endpoints, peripheral metabolites encode pathway-level states (bioenergetics, lipid remodeling, nucleotide turnover) that track aggressiveness across histotypes and stages. However, these pathway-level states are not necessarily tumor-type specific, since similar metabolic programs may arise from different oncogenic drivers or from non-malignant inflammatory, hormonal, or metabolic conditions. Therefore, their interpretation requires clinical context, appropriate control groups, and, when possible, integration with genomic, transcriptomic, proteomic, and imaging data. Furthermore, each biofluid presents specific limitations: serum and plasma may dilute tumor-derived signals, urine is strongly influenced by renal function, hydration, diet, and microbiome-derived metabolites, while ascites and ovarian cyst fluid are more proximal but not available from all patients. The following sections summarize the main evidence linking specific metabolic alterations in biofluids to clinically relevant features of EOC (Figure 2).

### 5.1. Tumor-Driven Lipid Alterations in EOCs

Lipids represent one of the most extensively and consistently reprogrammed metabolic classes in OC, reflecting profound alterations in FAO, membrane phospholipid remodeling, and bioactive lipid signaling that collectively sustain tumor growth, invasion, and therapeutic resistance.

Lipidomic profiling of preoperative serum samples from 147 patients with HGSC and 98 non-malignant controls enabled robust identification of tumor-associated lipid alterations associated with HGSC. In serum, HGSC is characterized by a global reduction in phospholipids, particularly phosphatidylcholines and lysophosphatidylcholines, along with decreased levels of sphingomyelins and cholesteryl esters [156,157]. This lipid-depleted signature is detectable across disease stages but becomes more pronounced in advanced tumors and appears largely independent of body mass index (BMI) or nutritional status, suggesting a tumor-driven systemic metabolic effect [156]. In contrast to this overall lipid depletion, alterations in circulating ceramides and triacylglycerols are strongly dependent on fatty acyl chain composition. Ceramides containing 16:0, 18:0, 20:0 and 24:1 fatty acids are consistently increased, whereas species with very-long saturated chains (23:0 and 24:0) are reduced. Triacylglycerol profiles show a parallel chain-length-dependent pattern, with depletion of short-chain species and preservation or enrichment of long-chain species, while saturation status exerts a minor effect. In contrast, diacylglycerol levels do not display consistent alterations [156,158].

Further insight into lipid metabolic reprogramming in OC is provided by a metabolomic study of pelvic and ascitic fluid, which reveals coordinated increases in metabolites associated with fatty acid transport, phospholipid remodeling and inflammatory lipid signaling [159]. The study cohort comprised 15 OC patients spanning FIGO stages I-IV, predominantly represented by plasmacytoid adenocarcinomas, with a smaller contribution from EC and CCC subtypes; a substantial proportion of cases presented with lymphatic metastasis and included both relapsing and non-relapsing disease, allowing metabolic features to be evaluated across different stages and clinical behaviors. Specifically, EOCs samples show elevated levels of palmitoylcarnitine, together with increased phosphatidylethanolamine (PE) and lysophosphatidylethanolamine (LPE) species (including PE 18:1/18:2, LPE 22:6, LPE 18:0, LPE 18:1 and LPE 22:1), as well as multiple lysophosphatidylcholine (LPC) species (LPC 24:1, LPC 22:4, LPC 20:1), consistent with enhanced phospholipase-driven membrane turnover/remodeling [159]. In contrast, lipoamide concentrations are reduced, suggesting diminished local antioxidant capacity in the EOC microenvironment. The same study identifies a marked upregulation of citraconic acid, which links OC-associated metabolic reprogramming to the *ACOD1*-itaconate axis. Citraconic acid, a highly electrophilic itaconate-related metabolite, can inhibit *ACOD1* activity, thereby modulating the conversion of cis-aconitate to itaconate, an anti-inflammatory metabolite, and acts as a potent activator of *NFE2L2* transcription factor [160,161]. *NFE2L2* activation promotes transcription of antioxidant and cytoprotective genes while concurrently restraining pro-inflammatory signaling, innate immune activation and cytokine production [162]. Overall, lipid-associated metabolites display robust discriminatory capacity, in several instances comparable to or exceeding that of established serum biomarkers such as CA125, and show significant associations with clinical stage, ascitic burden, lymph node involvement and disease recurrence, thereby linking lipid metabolic reprogramming to tumor aggressiveness and reinforcing its relevance in OC characterization [159].

Metabolomic profiling has identified hydroxybutyric acid metabolites and ketone bodies as prominent features of HGSC. In a large cohort including 158 patients with HGSC and 100 non-malignant controls, HGSC showed a consistent accumulation of 3-hydroxybutyric acid and related metabolites in serum [156,157], and were also associated with both disease presence and patient prognosis. These metabolites are thought to reflect alterations in redox balance and ketone-related carbon flux. Together, their systemic levels support the use of ketone body-linked metabolic patterns for outcome stratification in HGSC. This pattern is consistent with enhanced fatty acid β-oxidation and altered energy metabolism, further supported by increased acylcarnitines.

### 5.2. Other Tumor-Driven Metabolic Alterations as a Discriminator of Malignancy and Therapeutic Response in EOCs

Peripheral metabolite trajectories respond dynamically to EOCs as well as therapeutic approaches, enabling objective monitoring of tumor debulking and treatment impact across biofluids. A clear example comes from the study by Zhang et al. (2013) [163], which reported urinary MS-based metabolomic profiling in a well-characterized cohort comprising preoperative EOC patients, benign ovarian tumor (BOT) patients, healthy controls, and a smaller group of postoperative EOC patients. The preoperative EOC cohort included patients across FIGO stages I-IV, with a predominance of advanced-stage disease and elevated median CA125 levels, while BOT patients and healthy controls displayed substantially lower CA125 values and differed in age and menopausal status. Within the HGSC clinical framework, urinary metabolomic profiling highlights coordinated changes in nucleotide, amino acid, energy, and glycan metabolism that are closely linked to tumor presence. In particular, urinary nucleoside derivatives (including pseudouridine, N4-acetylcytidine and urate-3-ribonucleoside) are consistently increased in EOC compared with healthy controls and BOT, reflecting enhanced RNA turnover and activation of phosphoribosyl pyrophosphate-mediated nucleotide biosynthesis pathways. Parallel alterations are observed in energy metabolism, with elevated succinic acid suggesting increased mitochondrial activity and hypoxia-associated metabolic adaptation, and in amino acid metabolism, including reduced L-histidine and increased taurine, indicative of perturbed histidine and redox-related pathways. In addition, elevated levels of sialylated oligosaccharides (3′-sialyllactose and 3-sialyl-N-acetyllactosamine) point to dysregulated glycan metabolism, consistent with known mucin overexpression in OC. Importantly, several of these metabolites show partial normalization following surgical resection, supporting their association with tumor burden and reinforcing urine as a complementary, non-invasive biofluid for monitoring systemic metabolic consequences of OC.

Another urinary metabolomics study, including EOCs of mixed histological subtypes, predominantly HGSC but analyzed as a single group, together with BOTs and healthy controls, showed that urine-based metabolic profiles discriminate malignant from non-malignant disease [164]. Using a combination of LC separation techniques, this MS study identified increased urinary levels of nucleoside-related metabolites, particularly pseudouridine and its fragments, in OC patients compared with both control groups. Additional alterations involved amino acid- and energy-related metabolites, with increased hippuric acid, N-acetylglutamine and phenylacetylglutamine, as well as elevated creatinine and 5-phosphoribosylamine, indicating upregulation of amino acid handling. Although these findings support the diagnostic potential of urine-based metabolomics, their interpretation requires caution. Urinary metabolites are particularly sensitive to hydration status, renal function, diet, medication exposure, and gut microbial metabolism. Moreover, studies including mixed EOC histotypes or analyzing them as a single group may obscure subtype-specific metabolic patterns, especially when HGSC predominates. Larger cohorts with harmonized urine collection protocols and external validation are therefore needed before these signatures can be considered clinically actionable.

Concerning the standard chemotherapy regimen, DESI-MS-based spatial metabolomics adds mechanistic resolution by distinguishing epithelial versus stromal metabolic programs, linking lower chemotherapy response to epithelial pyrimidine metabolism enrichment and stromal hypoxia-associated metabolite accumulation (e.g., succinate, taurine). This implies that a given ‘LB’ signal might reflect the aggregate of spatially segregated resistant niches [165].

Finally, beyond metabolite panels, spectroscopic approaches applied to blood and urine have been explicitly discussed as capable of detecting chemotherapy impact on OC liquid biopsies, supporting the broader concept that treatment modality imprints measurable, longitudinal signatures in peripheral biofluids suitable for response assessment [166].

Together, these data support a multi-prognostic model in which serum lipid stress signatures (ceramide/triacylglycerol remodeling) and serum hydroxybutyrate-derivative patterns (systemic carbon/redox state) can be integrated with urine nucleoside markers (nucleotide turnover) to capture complementary dimensions of progression biology [163,167].

### 5.3. Metabolites Linked to EOC Therapy Resistance

Peripheral metabolite readouts in OC reflect therapy-driven selection pressures. They capture both tumor-specific metabolic alterations and metabolic changes driven by the stromal and immune microenvironment across multiple biofluids, including serum, plasma, urine, and ascites. When available, matched tissue-based metabolic mapping provides additional biological context. Recently, spatially resolved in situ metabolomics of HGSC using DESI-MS, showed that tumors from patients with poor or partial response to neoadjuvant chemotherapy exhibit enrichment of pyrimidine metabolism, particularly in the epithelial tumor regions (e.g., higher levels of uridine). In these same poorly responding tumors, the stroma displayed features consistent with hypoxia-associated metabolic remodeling, including increased levels of metabolites such as succinate and taurine, which together may reflect a microenvironment that promotes drug resistance [165]. In parallel, both systemic and local liquid compartments reflect platinum-driven metabolic vulnerabilities. Integrative analyses of OC metabolomics consistently report perturbations in glutathione and amino acid networks in serum, plasma, urine, and ascites, most prominently involving cysteine/cystine, glutamate, and glycine, which are directly required for glutathione synthesis, as well as methionine-related pathways linked to redox balance [5]. These alterations are consistent with the central role of thiol buffering and redox homeostasis in platinum detoxification.

From an immunotherapy perspective, ascitic fluid is particularly informative, as it contains both soluble metabolites and immune cells exposed to the same metabolic constraints. OC ascites is enriched in immunosuppressive metabolites, including lactate and products of tryptophan catabolism, which are associated with reduced T-cell proliferation and effector function. These findings indicate that peripheral metabolite patterns in ascites can mechanistically contribute to immune escape and ultimately undermine checkpoint inhibitor efficacy [168].

A prototypical axis connecting peripheral metabolites to immuno-resistance involves tryptophan catabolism, whereby indoleamine 2,3-dioxygenase activity promotes local tryptophan depletion and downstream kynurenine-mediated immunosuppression [169]. Notably, metabolomics surveys in OC also report altered kynurenine-related signatures in biofluids and tissues, reinforcing the plausibility that serum/plasma tryptophan-kynurenine balance could serve as an accessible pharmacodynamic readout in immunotherapy-oriented trials [5].

### 5.4. Histotype and Genotype Correlated Metabolites

Genotype and histotype imprint distinct metabolic phenotypes that could be back-inferred from peripheral metabolites, although the strongest evidence currently comes from tissue-based profiling across major epithelial EOC subtypes. In the most comprehensive subtype-resolved metabolomics dataset reported, CCC exhibited a distinct tissue metabolome compared with HGSC and EC, including pathway alterations in glycolysis and the pentose phosphate pathway, serine biosynthesis, cysteine metabolism, and reactive oxygen species-associated programs aligned with the intrinsic platinum resistance of late-stage CCC [170]. Importantly, within CCC, unsupervised metabolic subclusters did not correlate with *ARID1A* protein status or *PIK3CA* mutation status, emphasizing that common driver alterations do not translate into a single deterministic metabolic state. Thus, peripheral metabolite phenotyping may need to incorporate microenvironmental context and metabolic heterogeneity rather than relying solely on mutation status [170]. For HGSC, NAA has repeatedly emerged as a common abundant metabolite in tissues and ascitic fluid, and is now mechanistically framed in relation to *NAT8L*-dependent synthesis and microenvironmental secretion, providing a plausible metabolic surrogate feature that complements near-universal *TP53* alteration in HGSC, even when the mutation itself is not directly measured in LB assays [16,171]. In support of these findings, ascitic NAA levels were shown to positively correlate with FIGO stage and markers of invasiveness in OC patients, indicating that NAA accumulation in ascites fluid increases with disease advancement and may reflect a more aggressive phenotype in vivo [106].

Although largely tissue-based, these metabolic programs are directly relevant to peripheral metabolomics. Subtype-specific pathways, including cysteine/glutathione and pentose phosphate activity in CCC or NAA-related metabolism in HGSC, are expected to shape metabolite availability in circulation and ascitic fluid and can therefore inform histotype-aware interpretation of serum, plasma, and urine metabolomic data [170,171].

### 5.5. Host Metabolic Context

Systemic risk factors and host metabolic state can modulate peripheral metabolite baselines and therefore confound or, if modeled explicitly, refine metabolite-based OC risk stratification and monitoring. Serum lipidomics in HGSC found that survival-associated lipid changes were not correlated with BMI in the subset with nutritional data, whereas several sphingomyelins and LPCs correlated with patient-reported food intake, indicating that dietary or nutritional status can measurably shape circulating lipid readouts even when broad anthropometrics do not track the prognostic lipid signal [167]. More broadly, systematic reviews of metabolomics in OC indicate that most biomarker discovery studies use serum, plasma, and urine samples. These studies consistently identify alterations in lipid and amino acid pathways, including FAO signatures and nucleotide-related metabolites [5]. Notably, these metabolic axes are also strongly influenced by obesity, insulin resistance, and related cardiometabolic risk states. This overlap underscores the importance of risk-factor aware normalization when translating peripheral metabolite panels into screening or surveillance tools [5]. In practical terms, for a precision-medicine workflow, these observations argue for integrating clinical covariates of host metabolism (dietary intake proxies, metabolic syndrome components, medication used) alongside biofluid metabolomics, to distinguish tumor-associated shifts (e.g., postoperative drop in urinary modified nucleosides) from host-driven variability in lipid and amino acid pools [163,167].

## 6. The Potential and Limitations of AI and Machine Learning in Translational Application

The growing availability of metabolomic datasets has fostered increasing interest in the application of artificial intelligence (AI) and machine learning (ML) approaches for biomarker discovery, disease classification, and predictive modeling. These methodologies are particularly attractive in metabolomics because they can assist in handling complex datasets characterized by large numbers of interconnected variables, facilitating feature selection, pattern recognition, and the identification of biologically relevant metabolic signatures [172,173]. However, despite their considerable potential, the implementation of ML-based approaches requires careful methodological consideration to ensure robustness, reproducibility, and clinical relevance.

A major challenge in metabolomics stems from the large number of measured variables relative to the size of most available cohorts. This imbalance can complicate model development and increase the likelihood of identifying patterns that may not generalize beyond the training dataset [172,173]. Consequently, feature selection represents a critical step in metabolomics-based ML workflows. Approaches such as LASSO regression, Random Forest, and Partial Least Squares Discriminant Analysis (PLS-DA) are commonly employed to identify the most informative metabolic features and reduce data complexity while preserving predictive performance [172,173,174]. Furthermore, the use of nested cross-validation is recommended to evaluate the robustness of models built on small cohort sizes [172]. The translational value of ML-derived metabolomic signatures ultimately depends on their ability to perform consistently across independent datasets. While many studies report encouraging results in internally validated cohorts, external validation remains essential for assessing model generalizability and clinical applicability [173,175]. Independent evaluation across different populations, analytical platforms, and study settings may help determine whether identified metabolic signatures reflect reproducible biological phenomena rather than dataset-specific characteristics. In this context, reporting frameworks such as the DOME (Data, Optimization, Model, Evaluation) recommendations [176] provide useful guidance for improving transparency in model development, evaluation, and reporting practices [175], since analytical variability, batch effects, and instrument-related fluctuations remain important sources of bias that require careful consideration during model construction and validation [172].

Another important consideration concerns model interpretability. Although advanced ML algorithms can achieve strong predictive performance, their adoption in biomedical research and clinical settings may be limited when the biological rationale underlying model predictions remains an unclear “black box” [172,173,175]. Recent efforts have therefore focused on the development of explainable machine learning approaches that enable the contribution of individual metabolites to be quantified and visualized [172,173]. Such strategies may facilitate the interpretation of predictive models and improve confidence in their biological plausibility. Moreover, integrating metabolomics-specific knowledge, including biochemical classification and pathway-level information, may provide additional context for interpreting model outputs and identifying metabolically meaningful disease-associated patterns [173]. Current ML applications in metabolomics are largely focused on single-omics datasets; however, increasing attention is being directed toward the integration of metabolomic information with other molecular, clinical, and imaging data sources [177]. Such integrative approaches may provide a more comprehensive representation of disease biology and support the identification of composite biomarker signatures with improved discriminatory performance. Nevertheless, successful clinical implementation will require not only methodological advances but also close collaboration among clinicians, computational scientists, and laboratory researchers to develop models that are robust, interpretable, and readily applicable in real-world clinical settings [172].

## 7. Conclusions and Future Perspectives

Tumor-associated metabolic reprogramming emerges as a central and functionally informative dimension of EOCs, particularly when interrogated through LB-based metabolomic profiling. Most currently available metabolomic evidence derives from HGSC cohorts, whereas data on other EOC histotypes remain comparatively limited. Across available studies and biological matrices, recurrent alterations in lipid, nucleotide, amino acid, and energy metabolism have been reported in EOC, reflecting the integrated effects of oncogenic lesions, microenvironmental constraints, and systemic host responses, although their reproducibility across independent cohorts and analytical platforms remains incompletely established. In contrast to single-analyte biomarkers, metabolomics captures these adaptations at a systems level, providing a dynamic and integrative representation of tumor biology that complements histological classification and molecular stratification. Among the affected metabolic axes, lipid metabolism is the most extensively and reproducibly remodeled. Coordinated changes in phospholipids, acylcarnitines, sphingolipids, bile acids, and inflammatory lipid mediators are detected across serum, urine, ascites, and tumor tissue, consistent with enhanced fatty acid uptake and oxidation, membrane remodeling, and bioactive lipid signaling. These processes are closely linked to tumor proliferation, invasion, and therapy resistance. Importantly, subsets of lipid-associated metabolites show strong discriminatory capacity between malignant and non-malignant conditions, in some cases approaching or matching the performance of established serum markers, highlighting their diagnostic potential. Parallel alterations in nucleotide and amino acid metabolism further reflect increased biosynthetic demand, accelerated RNA turnover, and redox adaptation. Several of these metabolites correlate with tumor burden, disease stage, and post-surgical dynamics, supporting the use of metabolomics not only for disease detection but also for the longitudinal monitoring of disease trajectory and therapeutic response. Evidence supporting the clinical applicability of metabolomic biomarkers is also emerging from prospective validation studies. The AminoIndex Cancer Screening (AICS) platform uses plasma-free amino acid profiles to generate multivariate risk scores for several malignancies, including uterine and ovarian cancers [178]. In a multicenter cohort of more than 5000 individuals, the uterine/ovarian AICS score provided proof-of-concept that circulating amino acid signatures can capture clinically relevant metabolic alterations associated with gynecologic malignancies. The decline in performance over longer follow-up periods suggests that circulating amino acid profiles may reflect ongoing tumor-associated metabolic perturbations rather than long-term cancer susceptibility. More broadly, the current evidence supporting metabolomic biomarkers in EOC should be interpreted with caution, as many studies remain limited by small cohorts, retrospective or single-center designs, heterogeneous patient populations, and the underrepresentation of non-HGSC histotypes and early-stage disease. Furthermore, many proposed metabolic signatures lack robust external validation, making it difficult to distinguish reproducible biomarkers from study-specific findings. Diagnostic performance may also be overestimated when comparisons are restricted to healthy controls rather than clinically relevant differential diagnoses, including benign adnexal masses, borderline tumors, endometriosis, inflammatory conditions, and other malignancies. Therefore, future studies should prioritize adequately powered multicenter cohorts, appropriate control groups, and standardized validation strategies.

Continued advances in analytical standardization, prospective validation, multi-omics integration, and AI-driven data analysis are expected to strengthen the clinical utility of LB-based metabolomics in EOC. However, successful translation into routine practice will require reproducible, standardized, and clinically interpretable metabolic signatures that complement existing diagnostic and monitoring strategies. Rather than replacing established diagnostic and monitoring tools, LB-based metabolomics should currently be viewed as a complementary functional layer that may improve risk stratification, disease monitoring, and therapeutic decision-making when integrated with CA125, HE4, imaging, histopathology, and molecular profiling. The next step for the field is therefore not only the discovery of additional metabolite biomarkers, but the rigorous validation of reproducible, standardized, and clinically interpretable metabolomic signatures.

## Figures and Tables

**Table 1:** Clinicopathological features of the main EOC histotypes [7,8].

EOC Histotype		Key Clinical Features	Putative Cell of Origin and Pathogenesis	Major Genetic Alterations	Diagnostic Criteria
Relative Frequency	Age at Diagnosis				
High-grade serous carcinoma		Nonspecific symptoms, ascites is frequently observed, usually bilateral involvment, advanced stage at diagnosis (FIGO III–IV), CA125 frequently elevated but aspecific	Fimbrial epithelium of fallopian tube, often via STIC lesions, rarely from ovarian surface; marked genomic instability	*TP53* expression alteration and mutations (≈100%); homologous recombination deficiency: *BRCA1*, *BRCA2*, *RAD51C -D*, *BRIP1*; widespread copy-number alterations	Solid, papillary or cribriform architecture with marked nuclear atypia (>3-fold variation), high mitotic index and frequent necrosis; diffuse WT1 positivity and mutation-type p53 expression.
~75%	~65				
Low-grade serous carcinoma		Indolent symptomatic or asymptomatic course, ascites is variably present, often associated with serous borderline tumor (SBT)	Stepwise progression from serous borderline tumors	MAPK pathway alterations: *KRAS*, *NRAS*, *BRAF*; *CDKN2A* loss; wild-type *TP53*	Papillary or micropapillary architecture with low-grade atypia and low mitotic activity; frequent psammoma bodies; WT1 and ER positive with wild-type p53 expression.
~5%	~43				
Clear cell carcinoma		Unilateral, large mass (Ø13 cm), early stage at diagnosis (FIGO I-II), associated with paraneoplastic hypercalcemia and thrombosis	Endometrial-type epithelium; strong association with ovarian endometriosis; epigenetic and chromatin remodeling defects	*ARID1A* loss-of-function; *PIK3CA* mutations; rare *TP53* and MMR deficiency	Tubulocystic, papillary or solid growth composed of hobnail or clear cells (glycogen-rich cytoplasm); HNF1β, napsin A and PAX8 positive; WT1 and hormone receptor negative.
~5% (higher in Asia)	~56				
Endometrioid carcinoma		Often unilateral, large mass (Ø11 cm), early stage at diagnosis (FIGO I), favorable prognosis when early	Endometrial-type epithelium; frequently arises in ovarian endometriosis; molecularly heterogeneous	Alteration of *CTNNB1*, *PIK3CA*, *PTEN*, *KRAS*, *ARID1A*; MMR deficiency; *POLE* mutations	Confluent back-to-back endometrioid glands with stromal invasion and frequent squamous differentiation; ER/PR positive, WT1 negative, usually wild-type p53.
~10%	~55				
Mucinous carcinoma		Large mass, unilateral, early stage at diagnosis (FIGO I)	Uncertain, often progresses from mucinous borderline tumors, teratomas or Brenner tumours;	*KRAS* mutations; *CDKN2A* loss; *TP53* alterations; *ERBB2*/HER2 amplification	Invasive mucinous carcinoma with expansile or infiltrative/destructive invasion (≥5 mm) and intracellular mucin; CK7 positive, variable CK20/CDX2, usually WT1 negative.
~5%	~55				

Abbreviations: ARID1A, AT-rich interaction domain 1A; BRAF, B-Raf proto-oncogene; BRCA1/2, breast cancer gene 1/2; BRIP1, BRCA1-interacting protein 1; CA125, cancer antigen 125; CCC, clear cell carcinoma; CDKN2A, cyclin-dependent kinase inhibitor 2A; CDX2, caudal-type homeobox 2; CK7/CK20, cytokeratin 7/cytokeratin 20; CTNNB1, catenin beta 1 (β-catenin); EC, endometrioid carcinoma; EOC, epithelial ovarian carcinoma; ER, estrogen receptor; ERBB2/HER2, erb-b2 receptor tyrosine kinase 2/human epidermal growth factor receptor 2; FIGO, International Federation of Gynecology and Obstetrics; HGSC, high-grade serous carcinoma; HNF1β, hepatocyte nuclear factor 1-beta; KRAS, Kirsten rat sarcoma viral oncogene homolog; LGSC, low-grade serous carcinoma; MAPK, mitogen-activated protein kinase; MC, mucinous carcinoma; MMR, mismatch repair; NRAS, neuroblastoma RAS viral oncogene homolog; napsin A, novel aspartic proteinase of the pepsin family A; PAX8, paired box 8; PIK3CA, phosphatidylinositol-4,5-bisphosphate 3-kinase catalytic subunit alpha; POLE, DNA polymerase epsilon catalytic subunit; PR, progesterone receptor; PTEN, phosphatase and tensin homolog; RAD51C/D, RAD51 paralog C/D; SBT, serous borderline tumor; STIC, serous tubal intraepithelial carcinoma; TP53/p53, tumor protein p53; WT1, Wilms tumor 1; Ø, diameter.

## Data Availability

Not applicable.

## Supplementary Materials

The following supporting information can be downloaded at: https://www.mdpi.com/article/10.3390/diagnostics16131983/s1, Table S1: LB-based clinical trials in OC.

## Abbreviations

The following abbreviations are used in this manuscript:

OC(s)	Ovarian Cancer(s)
EOC(s)	Epithelial Ovarian Cancer(s)
HGSC	High-Grade Serous Carcinoma
LGSC	Low-Grade Serous Carcinoma
CCC	Clear Cell Carcinoma
EC	Endometrioid Carcinoma
MC	Mucinous Carcinoma
FIGO	International Federation of Gynecology and Obstetrics
STIC	Serous Tubal Intraepithelial Carcinoma
LB	Liquid Biopsy
HE4	Human Epididymis Protein 4
cfDNA	Cell-Free DNA
ctDNA	Circulating Tumor DNA
CTCs	Circulating Tumor Cells
miRNAs	Micro RNAs
circRNAs	Circular RNAs
lncRNAs	Long non-coding RNAs
EVs	Extracellular Vesicles
TEPs	Tumor-Educated Platelets
cCAFs	Circulating Cancer-Associated Fibroblasts
DLA	Diagnostic Leukapheresis
BMI	Body Mass Index
CSF	Cerebrospinal Fluid
BAL	Bronchoalveolar Lavage
MS	Mass Spectrometry
NMR	Nuclear Magnetic Resonance
LC	Liquid Chromatography
GC	Gas Chromatography
DESI-MS	Desorption Electrospray Ionization Mass Spectrometry
NGS	Next-Generation Sequencing
PCR	Polymerase Chain Reaction
TET	Ten-Eleven Translocation
TME	Tumor Microenvironment
FAO	Fatty Acid Oxidation
NAA	N-Acetylaspartate
PE	Phosphatidylethanolamine
LPE	Lysophosphatidylethanolamine
LPC(s)	Lysophosphatidylcholine(s)
