# Supplementary material for: Liquid Biopsy-Based Metabolomics in Epithelial Ovarian Cancer: Challenges, Methodological Advances and Translational Considerations
Source: Diagnostics (Basel). 2026 Jun 25;16(13):1983. doi: 10.3390/diagnostics16131983 (PMC13360296; doi:10.3390/diagnostics16131983)
Supplement: Supplementary file 1 [file diagnostics-16-01983-s001.zip › diagnostics-4247784-supplementary.pdf]

Table S1. LB-based clinical trials in OC

ClinicalTrials.gov ID,<br>Clinical trial title	OC histotype	LB sample	LB analyte	Main clinical aim	Study design,<br>Current status
NCT03155451<br>Plasma ctDNA Detection in Diagnosis of Epithelial Ovarian Cancer	EOC (histotype not specified)	Peripheral blood	Plasma ctDNA; methylation markers	Diagnosis / methylation biomarker development	Observational, Completed
NCT04651946<br>Cell-free DNA Methylation for Epithelial Ovarian Cancer	EOC (exact histotype not specified); benign ovarian tumor comparator cohort included.	Peripheral blood and tumor tissue	Cell-free DNA methylation in peripheral serum	Diagnosis / possible surveillance biomarker development	Observational, Recruiting
NCT05801263<br>ctDNA Methylation for Epithelial Ovarian Cancer	EOC (histotype not specified)	Peripheral blood	ctDNA methylation	Diagnosis / prognostic stratification	Observational, Recruiting
NCT03738319<br>Non-coding RNA in the Exosome of the Epithelia Ovarian Cancer	HGSC	Peripheral blood	Exosomal non-coding RNA (miRNA and lncRNAs)	Biomarker discovery / diagnosis	Observational, Recruiting
NCT05212779<br>Predicting the Risk of Ovarian Cancer Recurrence Using Circulating Tumor DNA to Assess Residual Disease	EOC (histotype not specified)	Peripheral blood and tumor tissue	Tumor-informed ctDNA MRD testing	MRD detection / recurrence surveillance	Observational, Active, not recruiting
NCT05027828<br>ctDNA as a Novel Biomarker of Treatment Efficacy in Patients With Ovarian Cancer	HGSC	Peripheral blood	ctDNA	Treatment efficacy monitoring / MRD kinetics	Observational, Recruiting
NCT05446545<br>Bespoke ctDNA Assay for Recurrence and Treatment Response Monitoring in Advanced Epithelial Ovarian Cancer	Advanced epithelial OC: newly diagnosed cases after primary debulking surgery and platinum-sensitive relapsed EOC	Peripheral blood and tumor tissue	Bespoke ctDNA assay	MRD / recurrence prediction / treatment-response monitoring	Observational, Recruiting
NCT05931055<br>The Effectiveness of Liquid Biopsy in Differential Diagnosis and Early Screening of Epithelial Ovarian Cancer	EOC (histotype not specified)	Peripheral blood	cfDNA-based liquid biopsy / fragmentomics-style blood assay	Differential diagnosis / early screening	Observational, Not yet recruiting
NCT07439328<br>ASTRA Study: Circulating Tumor Cells in Blood of Patients With High-Grade Serous Ovarian Cancer	HGSC: FIGO stages III-IV	Peripheral blood	Circulating tumor cells (CTCs)	Diagnostic / translational biomarker study around primary surgery	Observational, Completed
NCT03302884<br>Circulating Tumor DNA as an Early Marker of Recurrence and Treatment Efficacy in Ovarian Carcinoma (CIDOC)	OC (histotype not specified)	Peripheral blood and tumor tissue	ctDNA	Recurrence detection / treatment efficacy monitoring	Observational, Completed
NCT03614689	OC (histotype not specified)	Peripheral blood and tumor tissue	ctDNA plus immune repertoire profiling	MRD characterization / hereditary-spectrum and immune-repertoire correlation	Observational, Active, not recruiting

Assessment of the Minimal Residual Disease in Ovarian Cancer From Circulating Tumor DNA and Immune Repertoire					
NCT05458973 Circulating Tumor DNA and BRCA Reversion Mutation in Advanced or Recurrent Ovarian Cancer Patients With Germline Mutation	Advanced or recurrent OC with germline BRCA mutation (exact histotype not specified)	Peripheral blood	ctDNA (BRCA reversion mutations)	PARP inhibitor resistance / BRCA reversion monitoring	Observational, Recruiting
NCT06071286 Sequential profiling of tumor-derived circulating cell-free DNA (ctDNA) in advanced ovarian cancer patients (SPEED)	Advanced OC (histotype not specified)	Peripheral blood	Tumor-derived circulating cell-free DNA / ctDNA	Recurrence/progression detection and longitudinal genomic characterization	Observational, Recruiting
NCT04022863 Ovarium Cancer Detection by TEP's and ctDNA	OC (histotype not specified)	Peripheral blood	Tumor-educated platelets (TEPs) and ctDNA	Preoperative diagnosis / early detection	Observational, Recruiting
NCT05801276 ctDNA Methylation for Detecting Ovarian Cancer	OC (histotype not specified)	Peripheral blood	ctDNA methylation	Diagnosis	Observational, Recruiting
NCT03691012 Circulating Tumour DNA as a Marker of Residual Disease & Response to Adjuvant Chemotherapy in Stage I-IV Ovarian Cancer	Stage I-IV OC (histotype not specified)	Peripheral blood	circulating tumour DNA (ctDNA)	Residual disease assessment and response to adjuvant chemotherapy	Observational, Active, not recruiting
NCT05763511 Improved Diagnosis of Ovarian Cancer by Use of Circulating Tumor DNA as a Biomarker	Patients with suspected OC (histotype not specified)	Peripheral blood	circulating tumour DNA (ctDNA)	Diagnostic accuracy (preoperative differentiation benign vs malignant masses)	Observational, Recruiting
NCT05976932 Circulating Tumor DNA Monitoring in Platinum-resistant Ovarian Cancer	HGSC (platinum-resistant)	Peripheral blood	circulating tumour DNA (ctDNA)	Treatment response monitoring (consistency with imaging and CA125)	Observational, Not yet recruiting
NCT02178462 Biomarkers for Gynaecologic Cancer	Gynaecologic cancers including OC (histotype not specified)	Peripheral blood	Metabolomics (amino acid profiling; AminoIndex®)	Biomarker discovery / diagnostic discrimination (cancer vs benign/healthy)	Observational, Completed
NCT06558019 Exosome-based OCS Scores for Predicting Ovarian Cancer Recurrence	OC (histotype not specified)	Not explicitly stated (likely peripheral blood)	Exosome-based	Recurrence prediction / post-treatment surveillance	Observational, Not yet recruiting
NCT02431559 Circulating Tumor DNA in Patients With Ovarian Cancer	OC (histotype not specified)	Peripheral blood	ctDNA	Disease monitoring / ctDNA dynamics	Observational, Completed
NCT02839707 Circulating Tumor DNA in Gynaecologic Malignancies	Gynaecologic cancers including OC (histotype not specified)	Peripheral blood	ctDNA	Biomarker evaluation / monitoring	Observational, Recruiting
NCT04498117 Blood-based Genomic Profiling in Ovarian Cancer	OC (histotype not specified)	Peripheral blood	cfDNA / ctDNA	Genomic characterization	Observational, Recruiting

NCT01844986<br>ARIEL2: Rucaparib in Ovarian Cancer	HGSC (BRCA-mutated/HRD)	Peripheral blood	ctDNA (BRCA reversion)	Treatment efficacy / biomarker analysis	Interventional, Completed
NCT01968213<br>ARIEL3: Rucaparib Maintenance Treatment	Recurrent EOC	Peripheral blood	ctDNA / genomic biomarkers	Maintenance therapy efficacy	Interventional, Completed
NCT02470585<br>SOLO2: Olaparib Maintenance	BRCA-mutated OC	Peripheral blood	ctDNA (exploratory endpoints)	Maintenance therapy	Interventional, Completed
NCT01874353<br>NOVA: Niraparib Maintenance Trial	Recurrent OC	Peripheral blood	Circulating biomarkers (incl. DNA)	Maintenance therapy	Interventional, Completed
NCT03430836<br>Circulating microRNA Biomarkers in Ovarian Cancer	OC (histotype not specified)	Peripheral blood	Circulating miRNA	Biomarker discovery	Observational, Recruiting
NCT03015003<br>Extracellular Vesicle Biomarkers in Ovarian Cancer	OC (histotype not specified)	Peripheral blood	Extracellular vesicles / RNA	Biomarker discovery	Observational, Recruiting
NCT02327468<br>Circulating Tumor Cells in Ovarian Cancer	OC (histotype not specified)	Peripheral blood	CTCs	Prognostic biomarker	Observational, Completed
NCT02421393<br>CTC-based Prognostic Evaluation in Ovarian Cancer	OC (histotype not specified)	Peripheral blood	CTCs	Prognostic evaluation	Observational, Completed
NCT03033693<br>Plasma Proteomics in Ovarian Cancer	OC (histotype not specified)	Peripheral blood	Proteomics	Biomarker discovery	Observational, Recruiting
NCT02024113<br>Serum Biomarkers in Gynaecologic Cancer	Gynaecologic cancers including OC (histotype not specified)	Peripheral blood	Proteomics / metabolomics	Biomarker discovery	Observational, Completed
